# Trisomy 13 with unusual histological features typically described in Beckwith-Wiedemann Spectrum

**DOI:** 10.4322/acr.2024.486

**Published:** 2024-05-08

**Authors:** Wilker Dias Martins, Elisa França Chaves, Flavia Cristina Gonçalves de Aquino, Sean Brasil de Oliveira, Isabela Dorneles Pasa, Bruno Guimarães Marcarini, Vitor Ribeiro Paes, Chong Ae Kim, Regina Schultz

**Affiliations:** 1 Universidade de São Paulo (USP), Faculdade de Medicina, Hospital das Clínicas, Divisão de Anatomia Patológica, São Paulo, SP, Brasil; 2 Universidade de São Paulo (USP), Faculdade de Medicina, Hospital das Clínicas, Instituto da Criança, Unidade de Genética, São Paulo, SP, Brasil; 3 Universidade de São Paulo (USP), Faculdade de Medicina, Departamento de Patologia, São Paulo, SP, Brasil

**Keywords:** Genetics, Aneuploidy, Autopsy, Pathology, Case Reports

## Abstract

Trisomy 13, known as Patau syndrome, is a common aneuploidy with a well-known clinical phenotype. This case report describes a trisomy 13 patient with unusual autopsy findings, including features resembling the Beckwith-Wiedemann Spectrum. Due to abnormalities of gestational ultrasounds, a prenatal karyotype of amniotic fluid cells was performed, which resulted in 47, XY+13. Autopsy microscopy studies identified leptomeningeal glioneuronal heterotopia, which was not described as belonging to Patau syndrome. Other atypical findings were diffuse hyperplasia of pancreatic islets of Langerhans and adrenals enlargement with marked adrenocortical cytomegaly, characteristically seen in the Beckwith-Wiedemann Spectrum. Molecular genetic tests were not performed for the Beckwith-Wiedemann Spectrum. Still, due to the rarity of both disorders, this report may support the evidence that trisomy 13 can affect tissue organization and lead to unusual histopathologic features resembling classic overgrowth disorders.

## HIGHLIGHTS

Functional potential of chocolate: Prebiotic, antioxidant, antidiabetic, good mood food, for Alzheimer’s disease (AD), anticancer, antihypertensive, and anti-inflammatory

Effect of processing on chocolate: Fermentation for brown cocoa colour and flavour precursors. Roasting affects polyphenol stability

Improvements in the functionality of chocolate: Fortification of chocolates with the addition of fruits e.g. mulberry, lemon powder, passion fruit seeds, and probiotic strains

## INTRODUCTION

Trisomy 13 (T13) is a common aneuploidy with a recognizable clinical phenotype characterized by the cardinal triad of orofacial clefts, microphthalmia, and postaxial polydactyly.^1^ Large structural genetic rearrangements such as aneuploidies lead to a complex chain of events during organogenesis, which still needs to be elucidated entirely more than 60 years after describing the Patau syndrome as a consequence of an extra numerary chromosome 13.^2^

Another condition associated with alterations in organogenesis is Beckwith-Wiedemann syndrome, the commonest genetic overgrowth disorder that affects about 1 in 10,500 lives. It's a common human genomic imprinting disorder whose diagnosis can be made by clinical features or molecular testing for genetic and epigenetic changes on the chromosome 11p15 region.^3^

This case report describes a T13 patient with unusual histological features typically described in Beckwith-Wiedemann Spectrum (BWSp).

## CASE REPORT

We report a case of a male newborn delivered at 37 weeks of gestational age by vaginal labor after four days of premature rupture of the ovular membranes. His birth weight was 2.226 g, and his APGAR score was 1/1/1. Prenatal karyotype had already been performed with amniotic fluid cells, resulting 47,XY+13, compatible with Patau Syndrome. At birth, the clinical findings were compatible with Patau Syndrome, including retrognathism, micrognathia, flattened and enlarged nose, long philtrum, low-set ears, microcephaly, omphalocele, postaxial polydactyly in both hands and feet and imperforate anus. The patient died 28 minutes after birth, and due to the lethal prognosis of the malformations, no invasive measures were performed (including complementary exams). The cause of death was signed as Trisomy 13.

He was the first child of a healthy, non-consanguineous couple. His mother was 16 years old and had no relevant previous history besides an atrial septal defect surgically corrected three years before the pregnancy. Due to her history of cardiopathy, her pregnancy was considered high-risk. She followed nine prenatal appointments, and her serologic tests were all negative for syphilis, HIV, hepatitis B, and she was susceptible to toxoplasmosis.

## AUTOPSY FINDINGS

**Figure 1:**
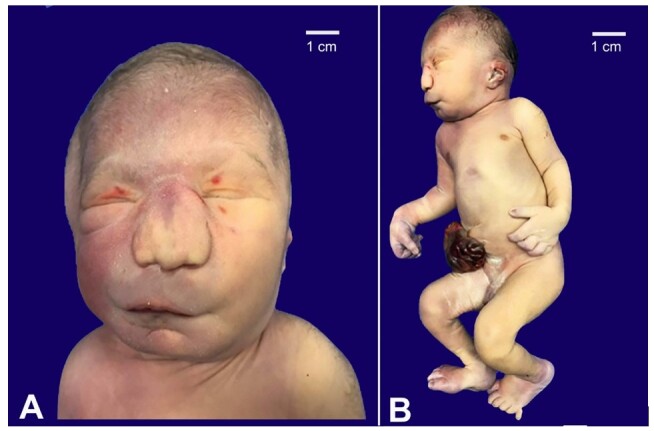
**A –** Ectoscopy of the corpse showing multiple facial dysmorphisms (hypotelorism, flat nose, long philtrum, micrognathia); **B –** Low-set and posteriorly rotated ears, polydactyly and omphalocele.

The body external examination revealed multiple facial malformations, including retrognathism, micrognathia, hypertelorism, flattened, enlarged nose and philtrum, and low-set ears (Figure 1A). Microcephaly was also attested. The abdominal inspection showed an omphalocele measuring 3.0 cm in its longest axis with herniation of intestinal loops (Figure 1B).

**Figure 2:**
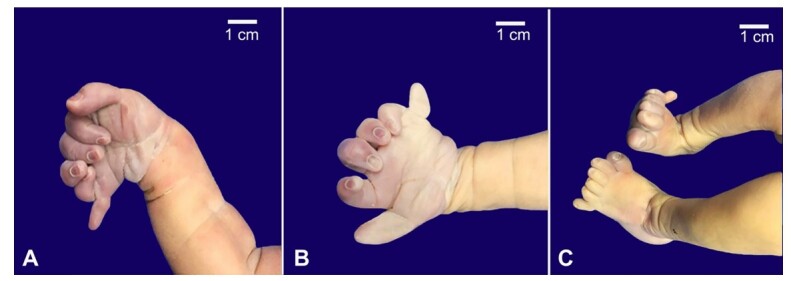
Postaxial polydactyly is observed in both the right (**A**) and left (**B**) hands and also in the feet (**C**).

The hands presented six-finger symmetric postaxial polydactyly (Figure 2). The anus was imperforate. No macroscopic malformations of the nervous system were observed.

**Figure 3:**
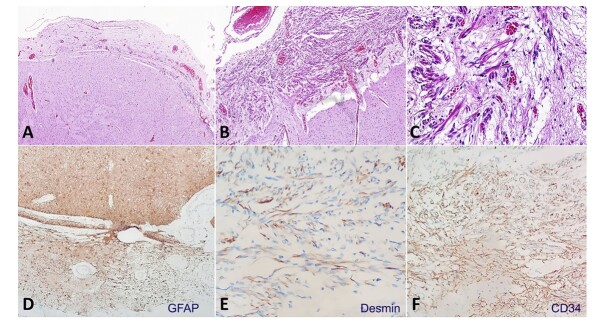
Photomicrography of the pons. **A –** Expansion and increase in cellularity of the arachnoid space (HE, 50X); **B –** Interruption of the glia limitans with herniation of glial cells into the arachnoid space (HE, 100X); **C –** Focal muscular differentiation and vascular proliferation (HE, 200X); **D –** Immunohistochemical profile reveals positivity of the glial cells to GFAP (100X); **E –** The cells also showed focal positivity for desmin (400X); **F –** CD34 highlights the vascular proliferation (200X).

The central nervous system microscopic examination revealed an interruption of the glia limitans in the pons topography, with glial cells that herniated into the arachnoid space, supported by the immunopositivity to GFAP, compatible with the diagnosis of leptomeningeal glioneuronal heterotopia, with a focal muscular differentiation and vascular proliferation of the adjacent tissue, supported by the positivity for desmin and CD34, respectively (Figure 3).

**Figure 4:**
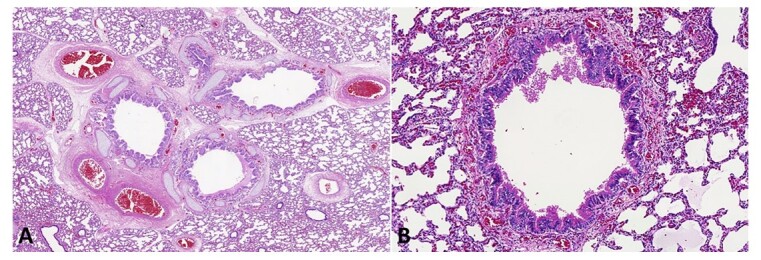
Photomicrographs of the lungs show bronchial tree malformation. **A –** Dilated bronchi with irregularly distributed cartilage (HE, 50X); **B –** Bronchiole unaccompanied by its pulmonary artery (HE, 100X).

At the thoracic cavity overture and cardiac dissection, an atrial septal defect and minimal dysplasia of the tricuspid valve were observed. The lungs weighed together 30.0 g (reference value: 43.6 g), less than expected for the age. At microscopy, a bronchial tree malformation was shown, characterized by dilated bronchi with irregularly distributed cartilage in addition to bronchioles unaccompanied by their pulmonary arteries or of very disproportionate size (Figure 4).

**Figure 5:**
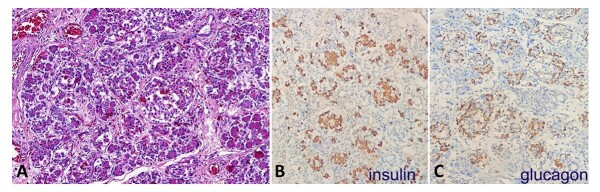
Photomicrographs of the pancreas. **A –** Increased number of islet cell clusters (HE, 100X); **B –** Increased immunohistochemical expression of insulin (200X); **C –** Similar findings in the immunohistochemical expression of glucagon (200X).

The testicles were located in the abdominal cavity, configuring a cryptorchidism. A Meckel's diverticulum measuring 2.5 × 2.0 cm in the small intestine was found. The pancreas weighed 6.0 g (reference value: 2.3 g), and the microscopy revealed an increased number of islet cell clusters, enhanced by immunohistochemistry, compatible with pancreatic adenomatosis (Figure 5).

**Figure 6:**
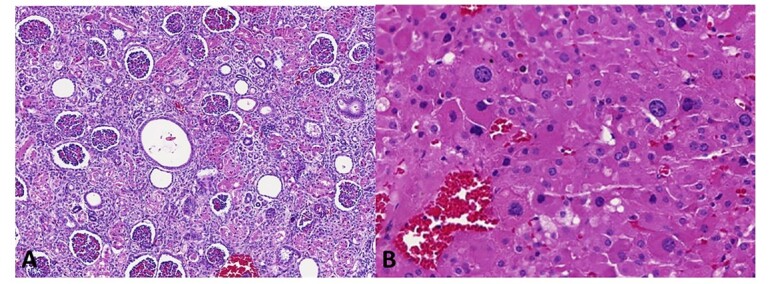
**A –** Photomicrography of the kidney showing tubular dilation and malformation of the tubular structures (HE, 100X); **B –** Photomicrography of the adrenal cortex showing karyomegaly and cytomegaly of the cells (HE, 400X).

Histologically, the kidneys showed focal tubular dilation with slight malformation of the tubular structures. The adrenals weighed 8.0 g together (reference value: 6.3 g), and the microscopic examination showed bilateral karyomegaly and cytomegaly of the adrenal cortex cells (Figure 6).

The remaining viscera were within normal limits. The placenta measured 16.0 × 14.0 × 2.0 cm and weighed 362.0 g. The histological analysis demonstrated villous immaturity for gestational age, chorangiosis, and intervillous hematoma. Chorionic membranes and umbilical vessels did not present any particularities.

## DISCUSSION

T13 (Patau syndrome) is the third most common aneuploidy in life-born infants, with an approximated birth prevalence of 1:18.000. Severe multiple congenital anomalies, including microcephaly, scalp defects, frequent holoprosencephaly, microphthalmia, orofacial clefting, congenital heart defects, postaxial polydactyly, severe developmental delay, and early mortality characterize it. Free T13 occurs in approximately 75% of all cases and is associated with advanced maternal age effect, with increased incidence of meiotic nondisjunction, mainly during meiosis I. Other etiologies are Robertsonian translocation and mosaic T13; banded chromosome study establishes the diagnosis. The mean life expectancy is 10 days, and more than 90% of the individuals die in the first year of life. Life-saving and life-prolonging medical interventions increased the survival rates.^1^

BWSp is a classic imprinting disorder with a very heterogeneous clinical presentation, ranging from individuals with isolated asymmetric overgrowth to cases with a much more exuberant phenotype, including generalized overgrowth, neonatal persistent hypoglycemia, macroglossia, anterior abdominal wall defects, and predisposition to embryonal tumors, especially Wilms tumor and nephroblastomatosis. Genetic or solely epigenetic abnormalities affecting the gene expression pattern within 11p15.5–11p15.4, made of differentially methylated regions (DMRs), are the recognized molecular mechanism for this syndrome.^3^

Although a precise diagnosis of BWSp is preferentially reached through molecular testing, the 2018 consensus statement^4^ proposes clinical criteria to guide clinicians in considering such a diagnosis. The cardinal features include macroglossia; omphalocele; lateralized overgrowth; multifocal and/or bilateral Wilms tumor or nephroblastomatosis; hyperinsulinism (lasting beyond one week and requiring escalated treatment); specific pathologic findings (adrenal cortex cytomegaly, placental mesenchymal dysplasia or pancreatic adenomatosis). The suggestive findings are birth weight >2 standard deviations above the mean; facial naevus simplex; polyhydramnios and/or placentomegaly; ear creases and/or pits; transient hypoglycemia (lasting less than a week); tumors (neuroblastoma, rhabdomyosarcoma, unilateral Wilms tumor, hepatoblastoma, adrenocortical carcinoma or phaeochromocytoma); nephromegaly and/or hepatomegaly; umbilical hernia and/or diastasis recti. A score ≥4 is required for a clinical diagnosis, but individuals with ≥2 points merit genetic testing.^4,5^ The main features of the two pathologies are summarized in Table 1.

**Table 1:** Features of Trisomy 13 and Beckwith-Wiedemann

Trisomy 13	Beckwith-Wiedemann
Microcephaly	Macroglossia
Scalp defects	Omphalocele
Holoprosencephaly	Lateralized overgrowth
Orofacial clefting	Hyperinsulinism
Postaxial polydactyly	Specific pathologic findings:
Congenital heart defects	a) adrenal cortex cytomegaly
	b) placental mesenchymal dysplasia
	c) pancreatic adenomatosis
	Multifocal and/or bilateral Wilms tumor or nephroblastomatosis

This case shows a newborn with Patau syndrome presenting unusual neurologic findings associated with pancreatic and adrenal autopsy uncoverings that are classically seen in BWSp.

In our case, we identified leptomeningeal glioneuronal heterotopia (LGH), which is defined by the presence of ectopic leptomeningeal aggregates of glial cells.^6^ LGH is related to several clinical manifestations, including refractory epilepsy and cognitive impairment.^7,8^ Recent studies have shown that ectopic aggregations of glioneuronal cells are formed by postmitotic neurons. Immunohistochemistry is an essential tool to elucidate the lineage of ectopic cells. The positivity to NeuN and GFAP supports the neural and glial origin.^6,9^ Further studies are necessary to establish a direct correlation between Patau Syndrome and LGH.

Another atypical finding was a pronounced and diffuse hyperplasia of pancreatic islets of Langerhans, which has been reported in a few cases of Patau Syndrome.^10^ It is currently known that neonates with T13 can present with hypoglycemia episodes right after birth, usually regarding transient relative hyperinsulinism that can occur due to prematurity as well as intrauterine growth restriction.^11^ Hyperplasia of islets of Langerhans is a cause of persistent and severe hyperinsulinemic hypoglycemia. Although classically in the form of focal hyperplasia, this adenomatosis is often associated with BWSp, an imprinting overgrowth disorder. However, there is insufficient evidence to determine whether this microscopy finding could be a coincidence or part of the T13 condition. Still, it may be more prevalent than previously thought, especially in the hard-to-control hypoglycemia cases of Patau Syndrome.^12^ The presenting case showed an enlarged pancreas during the macroscopy procedure, weighing approximately three times its normal weight. On hematoxylin and eosin (HE), it was histologically characterized by islets of Langerhans hyperplasia, confirmed by insulin and glucagon markers on immunohistochemistry.

The third puzzling feature found in the autopsy was the enlargement of adrenals with marked adrenocortical karyomegaly and cytomegaly. To our knowledge, there is only one previous case report of Patau syndrome associated with adrenals enlargement, and the authors did not describe a microscopy analysis that could assess the occurrence of adrenal cytomegaly.^12^ Again, these findings have long been associated with BWSp, to the point that adrenocortical cytomegaly was even considered a pathognomonic feature in the past.^13^ However, other studies have found it to occur in other cases of fetal distress, such as hypoxia, with a prevalence as high as 3% in newborn autopsies.^14,15^

## CONCLUSION

Patau syndrome has variable clinical, morphological, and histologic phenotypes; the pathophysiology underlying this variation remains not fully understood. Further genetic tests were not performed to eliminate the possibility of two diagnoses (T13 and BWSp) in the individual reported herein. Still, due to the rarity of both disorders, this report supports the evidence that T13 can affect tissue organization and lead to unusual histopathologic features resembling classic overgrowth disorders.
